# Dynamics of Lipid Transfer by Phosphatidylinositol Transfer Proteins in Cells

**DOI:** 10.1111/j.1600-0854.2008.00794.x

**Published:** 2008-08-06

**Authors:** Sadaf Shadan, Roman Holic, Nicolas Carvou, Patrick Ee, Michelle Li, Judith Murray-Rust, Shamshad Cockcroft

**Affiliations:** 1 Lipid Signalling Group, Department of Cell and Developmental Biology, University College LondonLondon WC1E 6JJ, UK; 2 Current address: Nature Publishing Group4 Crinan Street, London N1 9XW, UK; 3 Structural Biology Laboratory, London Research Institute, Cancer Research UK, Lincoln’s Inn FieldsLondon WC2A 3PX, UK

**Keywords:** lipid-binding cavity, lipid exchange, PITP domain, PtdCho transport, PtdIns transport

## Abstract

Of many lipid transfer proteins identified, all have been implicated in essential cellular processes, but the activity of none has been demonstrated in intact cells. Among these, phosphatidylinositol transfer proteins (PITP) are of particular interest as they can bind to and transfer phosphatidylinositol (PtdIns) – the precursor of important signalling molecules, phosphoinositides – and because they have essential functions in neuronal development (PITPα) and cytokinesis (PITPβ). Structural analysis indicates that, in the cytosol, PITPs are in a ‘closed’ conformation completely shielding the lipid within them. But during lipid exchange at the membrane, they must transiently ‘open’. To study PITP dynamics in intact cells, we chemically targeted their C95 residue that, although non-essential for lipid transfer, is buried within the phospholipid-binding cavity, and so, its chemical modification prevents PtdIns binding because of steric hindrance. This treatment resulted in entrapment of open conformation PITPs at the membrane and inactivation of the cytosolic pool of PITPs within few minutes. PITP isoforms were differentially inactivated with the dynamics of PITPβ faster than PITPα. We identify two tryptophan residues essential for membrane docking of PITPs.

Phosphatidylinositol transfer proteins (PITPα and β) are highly conserved 32-kDa soluble proteins that can bind and transfer phosphatidylinositol (PtdIns) and phosphatidylcholine (PtdCho) between cellular membranes *in vitro*(1–3). They are ideal candidates for the intracellular distribution of PtdIns from its site of synthesis, the endoplasmic reticulum (ER), to other membrane compartments. Mice deficient in PITPα die shortly after birth because of neurological defects, while PITPβ deficiency is embryonically lethal (4–6). PITPα is required for phospholipase-C- and phosphoinositide-3-kinase-mediated signalling during axonal outgrowth, whereas PITPβ participates in delivery of membrane vesicles and maintaining the actin ring at the cleavage furrow during cytokinesis (2,7–14).

The two human PITPs are 77% identical and 94% similar in amino acid sequence. Structurally, soluble PITPα and PITPβ are also very similar (15–17). An eight-stranded concave β-sheet flanked by two long α helices forms a hydrophobic cavity that protects a single phospholipid from the hydrophilic environment of the cytosol (Figure 1A). An α helix (G-helix), together with 11 C-terminal amino acid residues, functions as a ‘lid’ to close the cavity. In the crystal structure of apo-PITPα, structural changes including repositioning of the G-helix, C-terminal tail and the ‘lipid-binding loop’ (Figure 1B) suggest that the apo form may resemble the membrane-associated form. These changes would expose the hydrophobic lipid-binding side chains of apo-PITPα monomer, but interactions with a second PITPα molecule serve to bury these residues in the dimer interface (18). Also, PITPα dimerizes when its 24 C-terminus amino acids are proteolytically removed with subtilisin (19), a treatment that would expose at least some of its hydrophobic side chains. This PITPα dimer cannot participate in lipid transfer.

**Figure 1:**
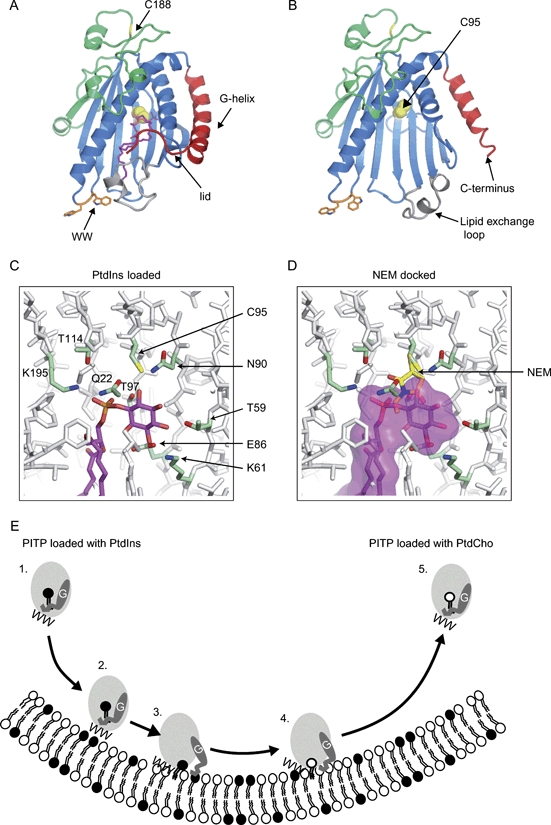
The C95 residue in PITPα is located close to the lipid-headgroup-binding site The position of C95 residue is shown in A) the closed (lipid-bound) and in B) the open (lipid-free) conformations of a PITPα molecule. The lipid-binding core residues are coloured blue, the G-helix and the extended 11 amino acids at the C-terminus that form the lid are coloured red, the regulatory loop is coloured green and the lipid exchange loop (18) is coloured grey. C95 is depicted as balls and is coloured yellow; it is inaccessible to small molecules in the closed conformation (A) but exposed in the apo structure (B). The backbone of the surface residue C188 is coloured yellow. In the apo structure, the lipid exchange loop and the G-helix have moved to the open configuration and the C-terminal region is disordered. The side chains of W203 and 204 are coloured orange. The diagrams were generated using the pymolsoftware with PDB files 1t27 and 1kcm. C) A stick model showing a PtdIns molecule (magenta carbon atoms) and the functionally important inositol-binding residues K61, N90, T59 and E86 in the lipid-binding cavity of PITPα. Also, labelled are the four residues that make contact with the phosphate moiety of the phospholipids, Q22, T97, T114 and K195. C95 (green carbon atoms and yellow sulphur atom) is seen to be in close proximity to the inositol ring (PDB code: 1UW5). D) Docking of NEM on C95 illustrates that alkylation of C95 would sterically hinder phospholipid binding. The NEM is shown with yellow carbon atoms and clashes with PtdIns (shown with a pink surface). E) Model for membrane interactions and lipid exchange by PITPs. 1) Soluble PITP bound to PtdIns in the closed conformation in the cytosol. 2) PITP initially docks onto a membrane using the two tryptophan residues (WW). 3) Conformational change of PITP at the membrane into an open form involves movement of the C-terminus and G α-helix, which exposes the hydrophobic surface of the lipid-binding cavity. This allows lipid exchange of PtdIns for PtdCho to occur. 4) Following lipid exchange, PITP bound to PtdCho undergoes a conformational change into the closed form. 5) PITP bound to PtdCho in the closed conformation is soluble and freely diffuses away from the membrane. PtdIns, solid circles; PtdCho, open circles.

The two lipid cargos of PITPs are PtdIns and PtdCho; while their headgroups are distinct, they occupy a similar location within PITPs (15,16). The five available hydroxyl groups of the inositol ring individually make contact with four amino acid residues of PITPα, T59, K61, E86 and N90 (Figure 1C) (We follow previous convention (16) and use the numbering of rat PITPα throughout the paper. Human PITPα has an almost identical sequence to rat PITPα with only a single residue deletion at position 52. This allows for easy comparison between this work and the previous publications), and mutation of any of these residues compromises PtdIns binding and transfer without affecting PtdCho binding and transfer (16). Although close to the inositol-headgroup-binding site (Figure 1C), C95 is not essential for lipid transfer as C95A mutation has no effect on lipid transfer ability of PITPα(20). However, chemical modification of C95 of recombinant PITPα*in vitro*with thiol-modifying reagents results in loss of PtdIns transfer activity (20), probably because the bound adduct occupies the space required by the lipid headgroup (Figure 1D). C95 is buried when PITPα is in the ‘closed’ soluble conformation but becomes exposed and prone to modification when PITPα is allowed to transiently associate with liposomes during lipid exchange (20).

Although PITPs can bind and transfer lipids between membrane compartments *in vitro*, whether they function as lipid transfer proteins *in vivo*has not been established. Analysis of cellular PITPs only identified the lipid-loaded forms (7,21), indicating the transient nature of the apo form. Thus, it is also unknown how frequently PITPs dock on membranes and undergo a cycle of ‘open’ and closed conformations, an indicator of lipid exchange *in vivo*(Figure 1E). In the case of PITPβ, a population is localized to the Golgi (22,23), but its conformational state is not known. Because thiol-modifying agents can only access C95 when PITPα is in the open conformation on liposomes (20), we used them – n-ethylmaleimide (NEM) and 2,2′-dithiodipyridine (DTDP) – as tools to measure the dynamics of PITP interaction with membranes in intact cells. Using the software tool mapas(24) to predict membrane-contacting protein surfaces, optimal docking area prediction on both PITPα and PITPβ identifies the dimer interface, with strong values for W203/W204 residues. We have tested this prediction *in vivo*and find that PITPα and PITPβ are unable to undergo a change to an open conformation at the membrane when the two tryptophan residues are mutated to alanine.

## Results

### PITP dynamics in live cells revealed using the sulphydryl-modifying reagent, NEM

**Figure 2:**
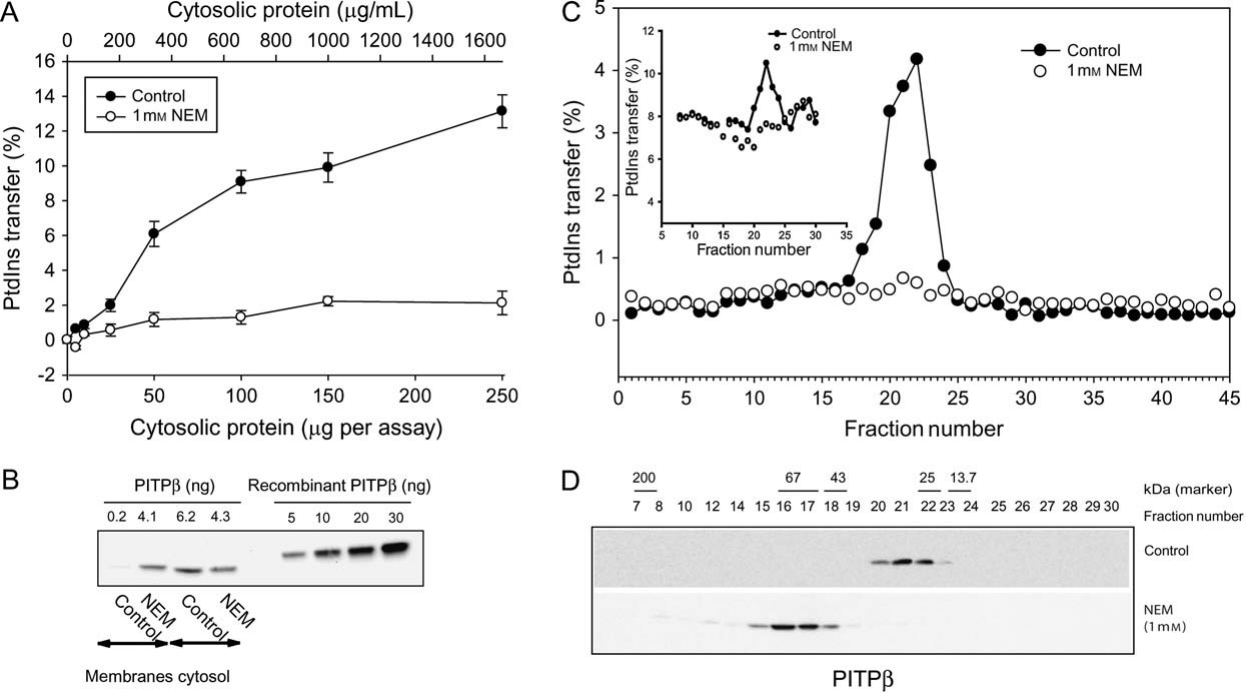
Inhibition of PtdIns transfer activity and membrane retention of PITPβ following NEM treatment of HL60 cells HL60 cells were treated with 1 mmNEM for 10 min at 37°C, and cytosol and membranes were subsequently prepared. A) PtdIns transfer activity in cytosol prepared from control (closed circles) and NEM-treated cells (open circles). B) Thirty micrograms of HL60 membranes and cytosol were analysed by SDS–PAGE, followed by western blot analysis from control and NEM-treated cells. Recombinant PITPβ was run in parallel to quantify the amount of PITPβ. The values are indicated above the blot in nanograms. C and D) HL60 cytosol (180 μL) from control and NEM-treated cells was fractionated by size exclusion chromatography (Superose 12 column 10/300), and the fractions were assayed for PtdIns and PtdCho transfer activity (inset) (C) and PITPβ distribution by western blot analysis (D). The column was calibrated using a kit containing proteins of molecular weight 200, 67, 43, 25, and 13.7 kDa, and their elution profile is indicated in (D). PITPβ in the cytosol prepared from NEM-treated cells elutes as a 67-kDa protein.

**Figure 3:**
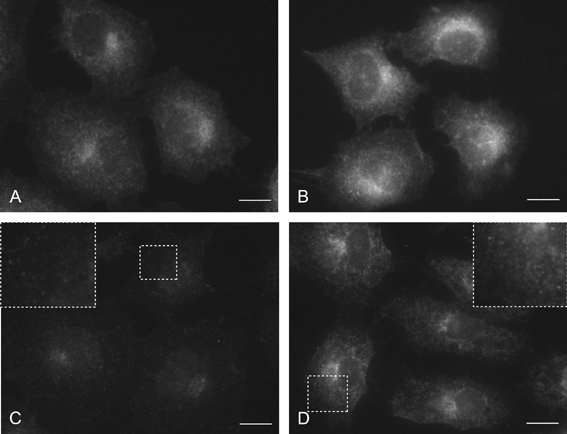
NEM treatment retains PITPβ at the Golgi and ER compartment in NRK cells NRK cells were treated with 100 μmNEM for 5 min, quenched with β-ME (20 mm) and either fixed in 4% paraformaldehyde before permeabilization with digitonin (40 μg/mL) (top panels) or permeabilized with digitonin on ice before fixation (bottom panels). Endogenous PITPβ was revealed by immunofluorescence using the rat-specific anti-PITPβ antibody 4A7. A) Cells were fixed first and then permeabilized. B) As (A) except that NRK cells were treated with 100 μmNEM for 5 min. C) cells were permeabilized with digitonin first on ice and then fixed and stained for PITPβ. D) As (C) except that NRK cells were treated with 100 μmNEM for 5 min. Bar scale: 10 μm.

Because NEM inactivation requires conformational changes of the protein upon binding a membrane and modification would prevent lipid exchange, inactivation of the transfer activity of cytosolic PITP provides an indication of PITP undergoing conformational change from closed to open. Intact HL60 cells were treated with NEM for 10 min and were then disrupted by sonication. The homogenate was centrifuged to separate membranes and cytosol. The cytosolic fraction of control cells has robust PtdIns transfer activity, which was inhibited in the cytosol prepared from NEM-treated cells (Figure 2A). The distribution of PITPβ between the membranes and the cytosol following NEM treatment of intact HL60 cells was also examined (HL60 cells predominantly express PITPβ (Figure S1). In control HL60 cells, PITPβ was mainly found in the cytosolic fraction. A very small amount of PITPβ that was occasionally found associated with the membranes probably corresponds to the Golgi-associated population of this isoform (Figures 2B and 3A). Following NEM treatment, a significant redistribution of PITPβ to the membrane fractions was observed (Figure 2B); PITPβ levels increased from 0.2 to 4.1 ng in the membranes (30 μg). This increase was accompanied by a slight decrease in the PITPβ content in the cytosolic fraction (Figure 2B). Based on densitometric analysis, we estimate that, upon NEM treatment, ∼10–15% of PITPβ became membrane associated taking into consideration the total amount of cytosolic and membrane proteins present. Therefore, retention of PITPβ to membranes alone cannot account for the inhibition of lipid transfer observed in the cytosol (Figure 2A).

Membrane association of PITPβ and inhibition of PtdIns transfer activity were dependent on both the concentration of NEM used to treat the HL60 cells and the time of incubation with NEM (Figure S2). Maximal retention to the membrane fractions was observed after treatment with 100 μmNEM for 10 min, a concentration that led to significant inhibition (∼40%) of lipid transfer. Nearly 75% of the transfer activity was lost with 250 μmNEM, and near maximal inactivation was observed with 500 μmNEM (Figure S2A,B). Treatment with 100 μmNEM for 2 min also caused significant membrane retention of PITPβ, and maximal retention was observed following treatment with 500 μmNEM for 2 min. Significant inhibition of lipid transfer (∼60%) was observed with 500 μmNEM (Figure S2C,D). In all, the association of PITPβ to membranes was rapid and was observed at much lower concentration of NEM than that required for inhibition of transfer activity.

We next examined whether PtdIns transfer activity observed in the cytosolic fraction was primarily because of PITPs. Cytosol from HL60 cells was separated by size exclusion chromatography, and the fractions were examined for both PtdIns and PtdCho transfer activity as well as by western blot using antibodies for PITPβ. A single peak of PtdIns (and PtdCho) transfer activity present in fractions 20–23 was observed (Figure 2C, see inset). Western blot analysis of the same fractions revealed that PITPβ (and PITPα, data not shown) eluted in these fractions (Figure 2D, top panel), establishing that the PtdIns transfer activity in HL60 cytosol is mainly because of the activity of PITPβ with a small contribution from PITPα (Figure S1). Cytosol was also prepared from NEM-treated HL60 cells and similarly fractionated by size exclusion chromatography. Minimal PtdIns or PtdCho transfer activity was observed in the fractionated cytosol from NEM-treated HL60 cells (Figure 2C). Western blot analysis of the same fractions revealed that NEM-modified PITPβ eluted as a protein with a larger than expected Stokes radius although on SDS–PAGE it still migrated as a 32-kDa protein, similar to PITPβ from control cells. Using molecular weight markers to calibrate the size exclusion chromatography column, we calculate that PITPβ elutes as a 67-kDa protein – that is, roughly twice its normal size. This can be rationalized by assuming that NEM-modified PITPβ exists in the hydrophilic environment of the cytosol as a dimer so that the hydrophobic residues that would otherwise be exposed in an apo-PITP-like structure are buried in the dimer interface.

### Visualization of PITPβ entrapment to the Golgi and the ER revealed using NEM

PITPβ has been reported to localize at the Golgi when examined by immunofluorescence (21–23,25). However, this observation does not identify whether PITPβ is constantly undergoing changes from closed to open conformation. We therefore used the rat kidney cell line normal rat kidney (NRK), which expresses significant levels of PITPβ (Figure S1), and examined localization of endogenous PITPβ upon treatment with 100 μmNEM in cells fixed prior to permeabilization (Figure 3A,B) and fixed after permeabilization (Figure 3C,D). PITPβ is localized at the Golgi but additionally shows a diffuse localization within the cytoplasm and faintly at the nuclear envelope (Figure 3A). This staining could correspond to PITPβ either associated with the ER or is cytosolic. When the cells are permeabilized prior to fixation, association of PITPβ with the Golgi observed by immunofluorescence is markedly reduced (Figure 3C). These results parallel the results when cells are homogenized and membrane fractions examined (Figure 2B). To examine whether PITPβ is on the ER membrane, we used low concentrations of NEM (100 μm) to capture PITPβ, followed by examination by immunofluorescence (Figure 3B,D). This NEM treatment does not affect the structure of the Golgi (Figure S3). In NEM-treated cells, PITPβ is now retained despite permeabilization of the cells prior to fixation (Figure 3D). In addition to the Golgi-localized PITPβ, the diffuse staining is still observed, indicating that PITPβ associates with the ER compartment as well (Figure 3D). Staining of the nuclear envelope is also observed (Figure 3D). When the cells were not permeabilized prior to fixation, NEM treatment enhanced the staining of the Golgi compared with non-treated cells (compare Figure 3A and 3B). In addition, the staining of the nuclear envelope is clearly observed (Figure 3B).

### NEM targets C95 of PITPβ, resulting in inhibition of PtdIns transfer

The thiol-modifying reagent NEM irreversibly alkylates the sulphydryl group on cysteine residues. In PITPα, only two of the four cysteine residues present in PITPα (C95 and C188) can be alkylated by NEM (20). A surface residue, C188, is always accessible to NEM, and its modification with NEM does not affect PtdIns transfer activity of PITPα(20). To demonstrate that NEM exerts its inhibitory effect by modifying C95 in PITPβ and PITPα, we mutated this residue to either alanine or threonine. C188 was also mutated to alanine. The mutant proteins were expressed in *Escherichiacoli*, and the purified recombinant proteins (Figure S4A,C) were examined for PtdIns transfer (Figure S4B,D). Wild-type (WT) PITPα and PITPβ and the corresponding mutants, C95A, C95T and C188A, all show comparable PtdIns transfer activity, indicating that C95 and C188 are not essential for PtdIns transfer (Figure S4B,D). When NEM was present during the assay, PtdIns transfer activity of both WT and C188A proteins was inhibited; in contrast, the mutants, C95A and C95T were resistant to inhibition by NEM (Figure S4B,D).

Perhaps more importantly, for both PITPs, inhibition of transfer activity only occurs provided that membranes are present at the time of exposure to NEM. Exposure of either membranes or PITPs to NEM prior to their use in the transfer assay did not inhibit lipid transfer, suggesting that NEM can only target the C95 residue in the presence of membranes (Figure S5A,B). These results are in agreement with earlier studies (20,26). Hence, NEM inhibits transfer activity of both PITPα and PITPβ, and C95 is the only residue involved in inhibition of PtdIns transfer. Furthermore, NEM can only access C95 when PITPα or PITPβ interact with membranes.

### Resistance to inhibition by NEM of the C95 mutants in COS-7 cells

**Figure 4:**
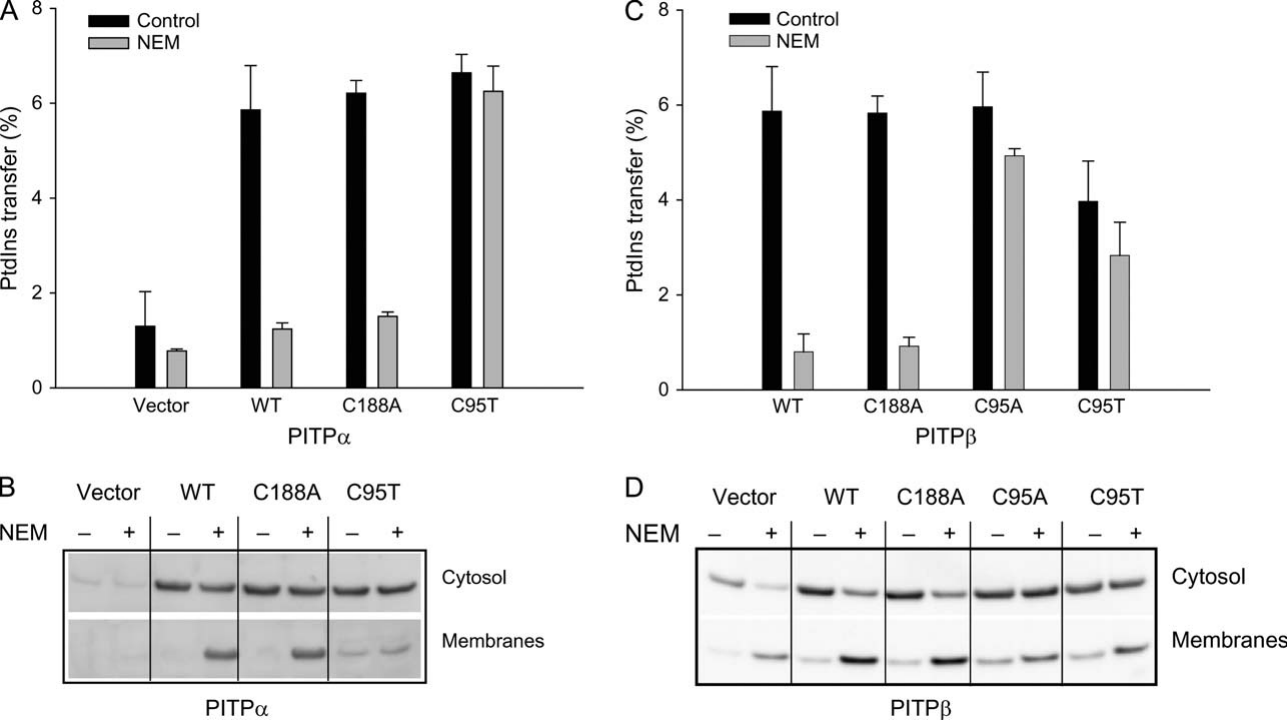
C95 mutants of PITPα and PITPβ expressed in COS-7 cells are resistant to inhibition by NEM A and B) COS-7 cells were transfected with vector-only cells, PITPα-WT and the mutants, C188A and C95T. The cells were treated with NEM (1 mm) for 10 min and were subsequently disrupted to obtain cytosol and membranes. A) PtdIns transfer was measured using 40 μg/mL cytosolic protein for 60 min at 25°C; B) PITPα was examined by western blot of cytosol and membranes from control and NEM-treated cells (50 μg protein per lane). C and D) COS-7 cells were transfected with PITPβ-WT and the mutants, C188A, C95T and C95A. The cells were treated with NEM (0.5 mm) for 10 min and were subsequently disrupted to obtain cytosol and membranes. C) PtdIns transfer was measured using 80 μg/mL cytosolic protein for 60 min at 25°C; D) PITPβ was examined by western blot of cytosol and membranes from control and NEM-treated cells (50 μg protein per lane).

We next examined whether the C95 mutants were resistant to inhibition by NEM in intact cells as the transfer activity of C95 PITP mutants is resistant to inhibition by NEM *in vitro*(Figure S4B,D). COS-7 cells were transfected with PITPα-WT and PITPβ-WT and their corresponding mutants, C188 and C95 mutants. Expression levels of PITPα-WT and its mutants were similar, and analysis of the western blots from four independent experiments indicated that PITPα expression was increased on average by 15-fold to 25-fold. By contrast, PITPβ expression was only twofold to threefold in three independent experiments. The cells were treated with NEM and were subsequently disrupted by sonication to prepare membrane and cytosol fractions. Transfer activity was monitored in cytosolic fractions of control and NEM-treated cells (Figure 4A,C). In cells overexpressing PITPα-WT or PITPβ-WT and the corresponding mutant C188A, NEM treatment led to inhibition of transfer activity. In contrast, transfer activity from cytosol prepared from cells expressing the C95 mutants of PITPα and PITPβ was resistant to NEM treatment (Figure 4A,C). C95A-PITPα was also examined in separate experiments and behaved similarly to C95T mutant (data not shown).

We also examined the retention of PITPα and PITPβ and their mutants to the membranes on NEM treatment. In Figure 4B (lower panel), both the PITPα-WT and the mutant C188A were retained at the membranes but not the C95T mutant. The cytosolic levels of PITPα and mutants were also examined, and a slight decrease in WT and C188A mutant was evident but not in C95T mutant (Figure 4B, upper panel). PITPβ was similarly examined for membrane retention upon NEM treatment. A robust retention of endogenous PITPβ is observed in the vector-only transfected cells as well as in cells expressing PITPβ-WT and C188A-PITPβ. In contrast, the C95 mutants are not retained on the membranes. The opposite situation is observed when cytosols were examined. While a prominent decrease in cytosolic PITPβ is visible in cytosol prepared from NEM-treated cells in vector-only cells, PITPβ-WT and the C188A mutant, the decrease in cytosolic C95 mutants is not very significant (Figure 4D, upper panel). Thus, mutation of C95 makes both PITPα and PITPβ resistant to attack by NEM so that the proteins are not retained at the membranes and the transfer activity of the proteins in the cytosol is unaffected.

### Two tryptophan residues define the membrane-contacting interface that is essential for membrane docking and transfer activity *in vivo*

**Figure 5:**
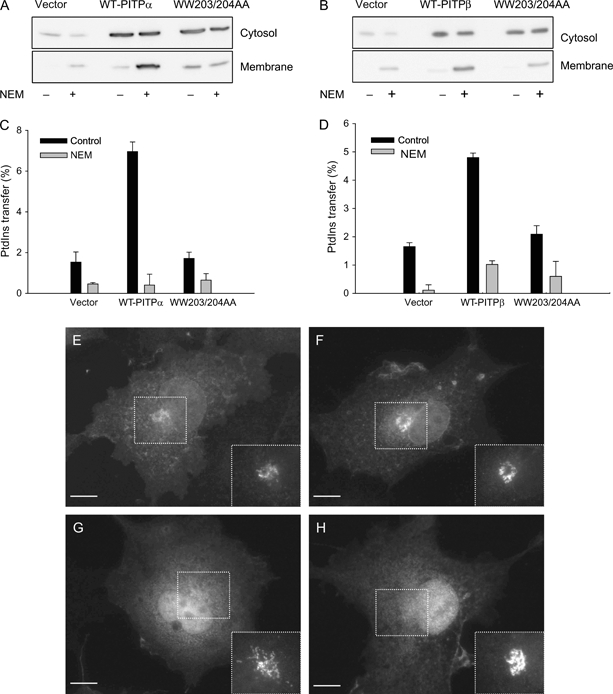
Mutation of tryptophan 203/204 in PITPα and PITPβ inhibits NEM-dependent retention to membranes and inhibits PtdIns transfer A–D) PITPα-WT and PITPβ-WT and their corresponding mutants (WW203/204AA) were transfected into COS-7 cells and treated with NEM (500 μm) for 2 min. A and B) Retention of PITPα and PITPβ to membranes by NEM is not observed when the two tryptophans are mutated to alanine. C and D) No increase in transfer activity is observed in cytosol prepared from COS-7 overexpressing the WW mutants of PITPα (C) or PITPβ (D). E–H) Overexpressed PITPβ-WT but not WW-PITPβ mutant is enriched at the Golgi upon NEM treatment. COS-7 cells were transiently transfected with PITPβ-WT (E and F) or the WW mutant (G and H). Forty-eight hours post transfection, the cells were treated with 100 μmNEM for 2 min (F and H). The insets show the staining of the Golgi using Giantin antibodies. Bar scale: 10 μm.

PITPs are present in the cytosol in their closed conformation and undergo an open conformation on the membrane surface to release its lipid cargo (Figure 1E). ATP is not required for this process. Computational analysis tools predict the importance of the tryptophan doublet W203-W204, conserved in both PITPs, for docking of PITP to membranes (Figure 1A,B), and we tested this possibility by mutating the two tryptophan residues to alanine in both PITPβ and PITPα. The WT proteins and the WW203/204AA mutants were expressed in COS-7 cells and treated with NEM to monitor membrane retention. Unlike PITPα-WT and PITPβ-WT (endogenous and overexpressed), the WW203/204AA mutants failed to associate with the membranes in the presence of NEM (Figure 5A,B). In addition, the mutant proteins were inactive for PtdIns transfer (Figure 5C,D), demonstrating the importance of these two residues for PITP docking to the membrane.

We also examined the intracellular localization of the PITPβ mutants in COS-7 cells. PITPβ-WT and the WW203/204 mutants in rats were transfected into COS-7 cells, and the cells were treated with 100 μmNEM for 2 min prior to fixation for immunostaining. (The monoclonal antibody (mAb 4A7) used in these experiments is rat specific for immunofluorescence and can therefore only detect the transfected proteins.) Overexpressed PITPβ-WT shows colocalization with Giantin, a marker for the *cis*Golgi (see insets). Staining of the nuclear envelope is also evident. As shown with endogenous PITPβ in NRK cells (Figure 3B), a significant increase in PITPβ-WT was observed at the Golgi upon NEM treatment (compare Figure 5E and 5F). In addition, prominent PITPβ-WT staining at the nuclear envelope, which is derived from the ER, was observed in the NEM-treated cells (Figure 5F). However, the WW203/204AA PITPβ mutant showed a more diffuse perinuclear localization that did not entirely overlap with the Golgi marker (Figure 5G), and indeed, in some of the cells, no perinuclear staining was observed. Furthermore no increase was observed at the Golgi or the nuclear envelope upon NEM treatment (Figure 5H). Instead, the WW mutant showed an increased nuclear localization in both control and NEM-treated cells (Figure 5G,H). Expression of the WW mutant does not affect the Golgi structure and neither does NEM treatment of COS-7 (see inset, Golgi staining with Giantin).

### Comparison of PITPα and PITPβ dynamics in intact PC12 cells

**Figure 6:**
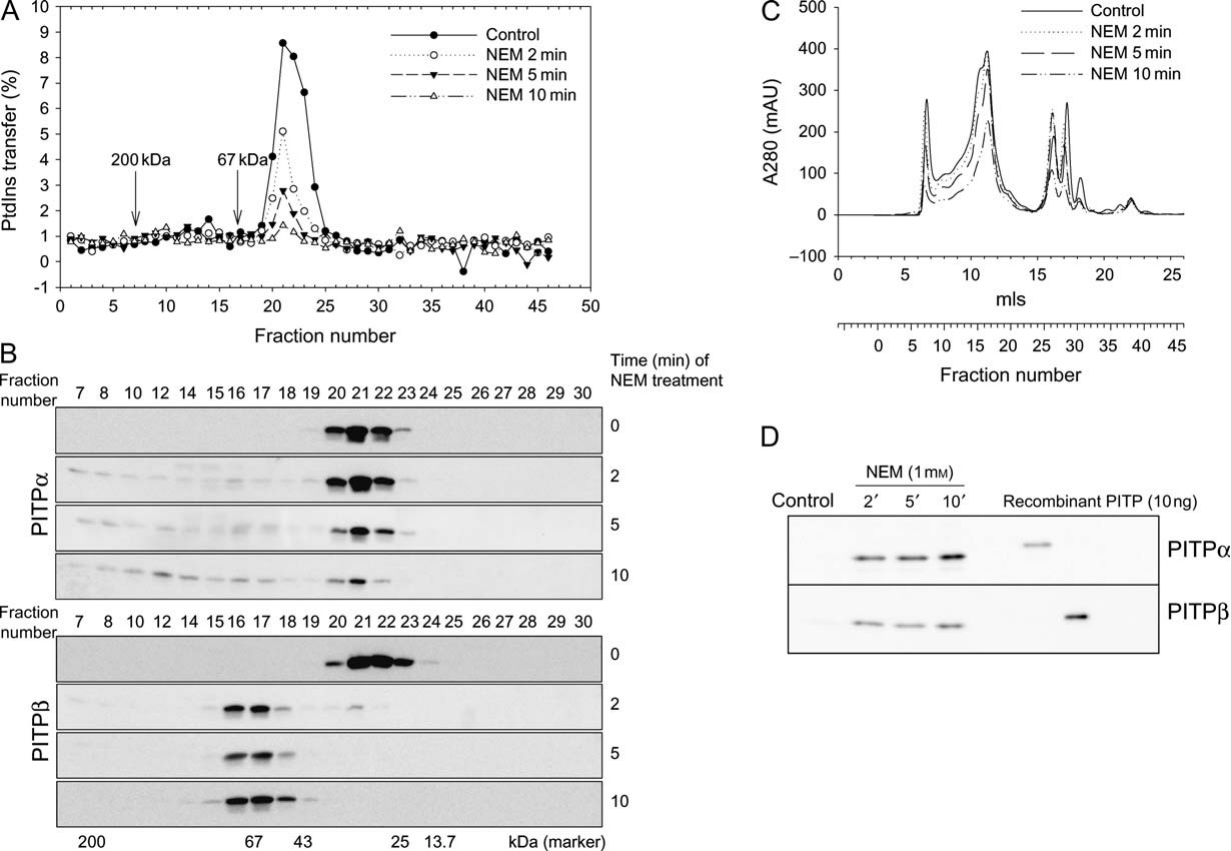
Differential inactivation of PITPα and PITPβ by NEM in PC12 cells Cells were treated with NEM for 2, 5 and 10 min, and the cytosol was subsequently fractionated by size exclusion chromatography. The fractionated cytosol was analysed (A) for PtdIns transfer activity and (B) by western blot analysis for their content of PITPα and PITPβ. C) The protein profile (optical density) of the cytosol fractionation showing protein recovery. D) Time-dependent retention of PITPα and PITPβ to membranes prepared from PC12 cells treated with 1 mmNEM.

To study the relative membrane dynamics of PITPα and PITPβ in intact cells, we surveyed various cell lines for the expression levels of PITPα and PITPβ; we identified PC12 as the cell line containing PITPα as its major PITP with significant quantities of PITPβ (Figure S1). PC12 cells were treated with 1 mmNEM for 2, 5 and 10 min, and the cytosols were fractionated by size exclusion chromatography. Transfer activity (Figure 6A), immunoreactivity of both PITPs (Figure 6B) and protein content (Figure 6C) were monitored in the individual fractions. (The amount of protein recovery is slightly less in cells treated with NEM for 10 min, and this underestimates the residual transfer activity in this sample.) In untreated cells, PtdIns transfer activity was coincident with the fractions containing both PITPα and PITPβ (fractions 20–24) (compare Figure 6A and 6B). In cytosol prepared from NEM-treated PC12 cells, PtdIns transfer activity was diminished with time of incubation but not totally obliterated (Figure 6A). Analysis of the same fractions by western blot revealed that although in NEM-treated cells, both PITPα and PITPβ now elute in earlier fractions on gel filtration (an indication of inactivation), the rate of inactivation was different (Figure 6B). Within 2 min of treatment with NEM, the majority of PITPβ now elutes in fractions 16–18. By contrast, a substantial amount of PITPα remains in fractions 20–23 at 2 min. Although there was a progressive reduction with time of PITPα in fractions 20–23 with a corresponding increase in immunoreactivity in the earlier fractions, even at 10 min, some PITPα still remained in fractions 20–23. This suggests that a population of PITPα remained unaffected by NEM treatment in the time frame of these experiments. This PITPα pool likely corresponds to the residual lipid transfer activity seen in these fractions (Figure 6A). In contrast to PITPβ, inactivated PITPα has a greater tendency to form oligomers/aggregates unlike PITPβ that mainly forms dimers (Figure 6B). In parallel, we also examined the retention of PITPα and PITPβ to membranes. In control PC12 cells, PITPα and PITPβ was not found in membranes, and upon NEM treatment, an increase in membrane-associated PITPα and PITPβ is evident within 2 min of NEM incubation (Figure 6D).

### Reversing thiol modification of C95 restores its lipid transfer activity

**Figure 7:**
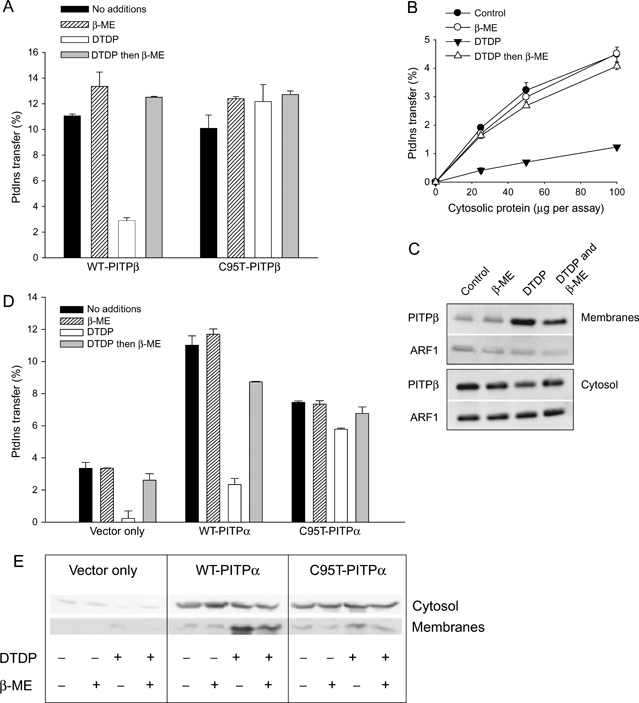
Inactivation of PITPα and PITPβ by DTDP and its reversal by β-ME A) Recombinant PITPβ-WT and the C59T mutant were incubated with DTDP (5 mm) for 10 min in the presence of liposomes. The proteins were examined immediately for transfer activity or were further incubated with β-ME (100 mm) for 10 min prior to monitoring lipid transfer. B and C) HL60 cells were treated with DTDP (1 mm) for 2 min, followed by β-ME (20 mm) for 10 min. B) Cytosol was analysed for PtdIns transfer and C) membranes and cytosol were examined for PITPβ content by western blot analysis. ARF1 was used as a loading control. D) COS-7 cells expressing PITPα-WT and the C95T mutant were treated with DTDP (1 mm) for 10 min and then with β-ME (20 mm) for a further 10 min. Cytosol (80 μg/mL protein) was examined for PtdIns transfer. E) Cytosol and membranes prepared from vector- and PITP-expressing cells treated with DTDP and subsequently with β-ME were examined for PITP retention to membranes (50 μg protein per lane).

The membrane-permeant oxidizing agent DTDP can also modify Cys residues, but unlike the NEM effect, this modification can be reversed by β-mercaptoethanol (β-ME). We found that, similar to NEM, *in vitro*DTDP can inactivate PITP transfer activity of recombinant PITP-WT but not the mutant C95T (Figure 7A). The DTDP-inactivated PITP protein was incubated with a 20-fold excess of β-ME for 10 min after which the protein was analysed for transfer activity. Transfer activity was recovered, indicating that modification of the C95 residue is reversible (Figure 7A).

We found that the transfer activity of endogenous PITP in cells could also be reversed after treatment with DTDP, HL60 cells were treated with DTDP for 2 min and were subsequently incubated for 10 min with β-ME. The cytosol was examined for PtdIns transfer activity, and it was found that, like NEM, DTDP pretreatment led to loss of transfer activity. Inhibition because of DTDP could be fully restored by β-ME (Figure 7B). Membrane retention of PITPβ was also evident in DTDP-treated HL60 cells, which was reversed when cells were further incubated with β-ME (Figure 7C). To confirm that C95 was the residue targeted by DTDP responsible for inhibition of transfer activity, C95T PITPα or PITPα-WT were expressed in COS-7 cells. The cells were then treated with DTDP for 10 min; to monitor reversibility, the cells were incubated for a further 10 min with a 20-fold excess of β-ME. Subsequent analysis of transfer activity from the cytosols of cells overexpressing PITP-WT and the C95T mutant showed that DTDP inhibited PITP-WT in both vector- and in PITPα-expressing cells. In contrast, transfer activity from the cells expressing the C95T mutant was resistant to inhibition by DTDP. The small inhibition observed is because of the presence of the endogenous PITPs present in the cells. Incubation of the DTDP-treated cells with β-ME led to recovery of the transfer activity in both vector- and PITPα-expressing cells. Membranes and cytosol were also examined by western blotting; Retention of both endogenous and the overexpressed PITPα-WT was observed upon DTDP addition but not of the C95T mutant. Incubation with β-ME led to a decrease in the amount of PITP-WT associated with membranes (Figure 7E).

## Discussion

The principal conclusion from this study is that, in intact cells, the lipid transfer proteins PITPα and PITPβ constantly interact with the membrane interface to exchange their lipid cargo (Figure 1E). Although structural analysis had implied that PITPs are exquisitely designed to bind and transfer lipids, and they certainly do so *in vitro,*it has not been previously possible to demonstrate that PITPs undergo lipid exchange in intact cells and if so, with what frequency. Only one previous study investigated the behaviour of PITP in cells using fluorescence lifetime imaging microscopy to examine fluorescence resonance energy transfer between green fluorescent protein-tagged PITP and BODIPY®-labelled PtdIns or PtdCho in COS-7 cells (27). There, it was found that interaction between PITP and PtdIns or PtdCho increases upon stimulation with EGF, and this occurred at 20 min after EGF treatment. In this experimental system, however, the basal dynamics could not be measured as the experiments were conducted in chemically fixed cells.

In this study, we used thiol-modifying chemicals (NEM and DTDP) to modify a strategically located cysteine residue as a tool to monitor interactions of PITP with membranes in the context of intact cells. Both PITPα and PITPβ contain four conserved cysteine residues at positions 95, 188, 192 and 231 in these proteins’ amino acid sequence. Previous *in vitro*studies showed that in PITPα, these four residues exhibit differential reactivities to thiol-modifying reagents such as NEM (20). For instance, C192 and C231 do not react with NEM, consistent with their side chains being buried in both the open and the closed lipid-bound structures. C188 is surface exposed and can be modified with NEM, but it is not functionally important. C95 is unique in that it is only reactive to NEM when PITP is associated with membranes, adopting an open conformation to release its lipid cargo (20). We show that the *in vitro*transfer activity of both PITPα and PITPβ is only inhibited when NEM is present during intermembrane transfer (Figure S5). The inhibition results from NEM modification of cysteine residue 95, which resides in the lipid-headgroup-binding cavity of PITP (Figure 1A–D); mutation of C95 to either alanine or threonine makes both PITPα and PITPβ resistant to inhibition by NEM and by DTDP (Figures 4 and 7).

Within 2 min of treating HL60 or PC12 cells with NEM, association of PITP with membranes is detected, indicating that, in intact cells, PITP molecules are constantly sampling membranes. It must be noted that, despite the increase in membrane association of PITPs upon NEM treatment, a significant proportion of these proteins are present in the cytosol (Figures 2B and 6B). This suggests one of two possibilities: the concentration of NEM used in this study is insufficient to result in the retention of the total cytosolic PITP pool to the membranes and hence their modification, or the PITP pool that has been covalently modified by NEM at the membranes is able to dissociate even without a bound phospholipid. The latter seems to be the case because the cytosolic pool of PITP from NEM-treated cells is inactivated for PtdIns transfer. Based on their loss of ability to transfer PtdIns and PtdCho, it appears that, within minutes of treatment with NEM, almost all the PITP molecules present in HL60, PC12 and COS-7 cells have been in contact with the membranes and thus had their C95 residues exposed to NEM. Our size exclusion chromatography data indicate that NEM-modified cytosolic PITPs elute as dimers. This would suggest that NEM binding has caused the PITP protein to adopt an open conformation, similar to that found in apo-PITP (18); we note that C95 in the apo-protein structure was reported as being chemically modified, as evidenced by an electron density peak 1.8 Å from the SG atom [modelled as a water molecule in the protein database (PDB) entry] (18). Apo-PITPα forms crystallographic dimers to bury the hydrophobic residues within the lipid-binding cavity in the dimer interface, and the two lipid exchange loops make contact with the partner lipid-binding regions. It has been suggested that binding of a phospholipid is required for PITPs to fully arrive at the closed soluble conformation (28,29). So, NEM-modified PITP likely adopts a dimeric arrangement analogous to that found in the apo-PITPα structure.

The open conformation NEM-modified PITPs are likely to dissociate from the membrane during lipid transfer more slowly than WT PITPs. Consequently, when membranes of NEM-treated cells are examined, some PITPs are detected that are still membrane associated. This is not seen in membranes prepared from control cells. When NEM-modified PITP eventually leaves the membranes, it forms dimers that shield its hydrophobic residues but are not capable of PtdIns transfer. Using NEM in intact cells, our data clearly demonstrate that the majority of PITPs have interacted with the membrane within 10 min. This emphasizes the dynamic nature of PITP interaction with membranes in intact cells.

When examined by immunofluorescence microscopy that both endogenous and overexpressed PITPβ localize at the Golgi compartment (21,23,27,30) as well as diffusely in the cytosol. The diffuse staining is because of PITPβ associating with membranous structures, most likely ER, as demonstrated by its retention in the cell following NEM treatment (Figure 3). As all the cellular PITPβ is inactivated upon NEM treatment, our data indicate that the Golgi pool of this protein is in rapid dynamic equilibrium with the ER pool. In addition, the membrane-associated PITPβ is undergoing a rapid change in conformation from open to closed.

In PC12 cells, a small fraction of PITPα appears to be somewhat resistant to inactivation by NEM in the 10-min time frame studied. There could be two explanations that are not mutually exclusive. The resistant subpopulation could represent the nuclear pool of PITPα that is not in close proximity to membranes and might not go through a cycle of open and closed conformation during the 10-min period studied in this study. Alternatively, it could represent a population of PITPα phosphorylated at Ser166, which causes inactivation of its PtdIns transfer activity by an unknown mechanism (31).

The first step in the process of lipid exchange is for PITP to dock on to membrane surfaces. We identify two tryptophan residues that are critical for this process in intact cells. We had previously shown that mutation of the two tryptophans to alanine in PITPα led to loss of PtdIns transfer activity *in vitro*(16). Using NEM as a tool for trapping the open form of PITP to membranes, a measure for docking can be monitored by assessing the retention of PITP to membranes. Mutation of these two tryptophan residues W203/W204 (Figure 1A) to alanine prevents membrane association of both PITPα and PITPβ. This is coupled to the loss of lipid transfer in these mutants indicating that, without docking to membranes, PITPs cannot transfer lipids.

We found that the WW203/204AA mutant of PITPβ is no longer enriched in the Golgi compartment and instead is localized in the nucleus. Although the reason behind the redistribution of this mutant within the cell is not clear, a possible explanation is that when WW203/204AA mutant of PITPβ is no longer retained at the Golgi, it is free to move to the nucleus. This is not surprising as PITPα, which is similar to PITPβ in both structure and size, also partitions into the nucleus (23). In the case of PITPβ-WT, because it preferentially localizes to the Golgi, the free population of it might be limited for it to also localize in the nucleus.

In summary, this study demonstrates a continuous sampling of the cellular membranes by PITPα and PITPβ making use of two tryptophan residues to initiate the ‘opening’ of the hydrophobic cavity. The two tryptophan residues are located at the end of the helix F adjacent to helix G that has to dislodge to open the cavity. Thus, by inserting the two tryptophan residues into the membrane, this would perturb the helix G. Tryptophan residues are known to have a preference for membrane surfaces because of the aromatic ring and its molecular shape (32,33). Our data support a model whereby PITPs undergo a cycle of closed and open conformation and are thus likely to function as lipid transfer proteins in intact cells. In the case of PITPβ, our results indicate that lipid transfer takes place between the ER and the Golgi.

## Materials and Methods

### Materials

Unless otherwise stated, all the tissue culture products used were from Invitrogen or Sigma. NEM and DTDP were prepared in dimethyl sulphoxide as stock solutions and kept in aliquots at −20°C. Once thawed, the aliquots were discarded. The anti-PITPα polyclonal antibody (Ab 674) and the anti-PITPβ monoclonal antibody (Ab 1C1) as well as the recombinant proteins (PITPα and PITPβ) were generated in-house (21). The monoclonal antibody made against rat PITPβ Ab 4A7 was used for immunofluorescence. Its specificity was verified using small interference RNA to deplete PITPβ.

### Culturing of HL60, PC12 and NRK cells

HL60 cells were grown in suspension in RPMI-1640 medium containing 12.5% heat-inactivated foetal calf serum (FCS), 50 IU/mL penicillin, 50 μg/mL streptomycin and 2 mml-glutamine. PC12 cells were grown in suspension in DMEM medium containing 10% heat-inactivated horse serum, 5% heat-inactivated FCS and 2 mmL-glutamine. NRK cells were cultured in DMEM medium containing 10% heat-inactivated FCS, 2 mml-glutamine, 50 IU/mL penicillin and 50 μg/mL streptomycin.

### Culture of COS-7 cells and transfection by electroporation

COS-7 cells were grown in DMEM supplemented with 10% heat-inactivated foetal bovine serum. The COS-7 cells (2 × 10^7^ cells) were trypsinized, washed and resuspended in 400 μL DMEM supplemented with 10% heat-inactivated foetal bovine serum. For electroporation (two pulses of 0.220 kV and 950 μF), the cells were mixed with 10 μg of the plasmid construct and 30 μg of Herring sperm carrier DNA. Cells were used 24–48 h post transfection.

### Mutagenesis and purification of recombinant PITPα and PITPβ proteins in *Escherichia coli*

PITPα and PITPβ were expressed using pRSETC vector (Invitrogen). Mutagenesis was performed using the QuickChange kit from Stratagene according to the protocol provided by the manufacturer. The constructs were all sequenced for verification that the mutations were introduced. The His-tagged proteins were expressed in the *E.* *Coli*strain, BL21(DE3)pLysS, and purified using nitrilotriacetic acid–agarose (Invitrogen) as described previously (34). The His-tagged proteins were desalted to piperazine-1,4-bis(2-ethanesulphonic acid (PIPES) buffer (20 mmPIPES, 137 mmNaCl and 3 mmKCl, pH 6.8) and stored at −80°C. The same primers were used to introduce the same mutations in PITPα and PITPβ in pcDNA3.1 vector for mammalian cell expression.

### Assays for PtdIns and PtdCho transfer activity *in vitro*

PtdIns transfer activity was assayed by measuring the transfer of [^3^H]PtdIns from radiolabelled rat liver microsomes to unlabelled synthetic liposomes (PtdCho:PtdIns ratio, 98:2 by molar percentage) as described previously (7). To examine the effect of NEM on recombinant proteins, PITPs were pretreated with NEM (0–5 mm) in the presence of liposomes and NEM was quenched with 20-fold excess of β-ME (20–100 mm) and then assayed for transfer activity. Percentage transfer was calculated from the total counts present in microsomes after subtracting the number of counts transferred in the absence of a PITP source. PtdCho transfer activity was monitored using permeabilized HL60 cells prelabelled with [^3^H]choline to label the choline lipids, predominantly PtdCho, exactly as described (30). Transfer activity was monitored in duplicate samples, and the error bars denote the range of the averages. For fractions obtained after size exclusion chromatography, individual fractions were analysed. All data presented are representative of at least three independent experiments.

### NEM treatment and preparation of membrane and cytosolic fractions

The protocol used for HL60, PC12 and COS-7 cells was essentially the same. Approximately 1–2 × 10^8^ cells were used per treatment. COS-7 cells were used either as adherent cells or trypsinized and used in suspension. The results were identical regardless of the protocol. Cells were suspended in 10 mL of HEPES buffer (137 mmNaCl, 2.7 mmKCl, 20 mmHEPES, 2 mmMgCl_2_, 1 mmCaCl_2_, 1 mg/mL glucose and 1 mg/mL BSA, pH 7.2) and treated with NEM or DTDP at 37°C at the indicated concentrations and times. NEM or DTDP treatment was terminated by the addition of 20 mmβ-ME or by centrifugation and washing with 10 mL of ice-cold PBS at 4°C. The cells were resuspended in 300 μL PBS in the presence of a protease inhibitor cocktail (Sigma). To prepare membranes and cytosol, the cells were sonicated on ice (50 microns, 3 × 15 seconds) and centrifuged for 10 min (2000 × ***g***, 4°C) to pellet the nuclei and unbroken cells. The lysate was centrifuged for 30 min at 110 000 × ***g***at 4°C to pellet the membranes, and the supernatant consisting of the cytosolic fraction was retained for further analysis. To remove contaminating cytosolic proteins, the membranes were resuspended in 1 mL of SET buffer (0.25 msucrose, 1 mmethylenediaminetetraacetic acid and 10 mmTris–HCl, pH 7.4), and centrifuged at 110 000 × ***g***(4°C, 30 min). The membranes were resuspended in 100 μL of SET buffer or in PBS. Protein concentrations were determined for both the membrane and the cytosolic fractions. The distribution of PITPs (α and β) between membranes and the cytosol was analysed through separation of the proteins on a 12% sodium dodecyl sulphate polyacrylamide gel followed by western blot analysis.

### Fractionation of cytosol by size exclusion chromatography and analysis of lipid transfer

For analysis of the cytosol by size exclusion chromatography, the cells were disrupted using repeated freeze–thaw cycles. This protocol was chosen so that the cytosol could be obtained in a small volume (150 μL). Two hundred millilitres of confluent HL60 or PC12 cells was used per condition, and the cell pellet was transferred into an Eppendorf tube and resuspended in 100 μL of PBS-containing protease inhibitor cocktail (Sigma). The cells were frozen at −80°C overnight. The following day, they were transferred to a salted ice bath for 6 h in a chilled cabinet, and then, they were transferred to −80°C overnight. This process was repeated on the second day. On the third day, the samples were rapidly thawed on ice and immediately centrifuged on a bench top Optima™ ultracentrifuge (Beckman) (110 000 × ***g***, 30 min, 4°C). The cytosol was carefully decanted and recentrifuged at 15 000 × ***g***for 10 min, and 150–180 μL (2–3 mg of total protein) was immediately loaded on a Superose 12 column 10/300; bed volume 24 mL (GE Healthcare) and eluted with PBS). 0.5 mL fractions were collected. (The first 3 mL was not collected; the conversion of elution volume to fraction number can be found in Figure 6C where both scales are shown.) Hundred-microlitre aliquots of the individual fractions were analysed for PtdIns or PtdCho transfer immediately, and 50-μL aliquots of the individual fractions were combined with sample buffer for western blot analysis. For PtdIns transfer, 150 μL of a mixture of microsomes and liposomes was added to each of the fractions and processed exactly as described previously (7). For PtdCho transfer, 100 μL of the fraction was analysed using the cytosol-depleted permeabilized HL60 cells as the donor compartment exactly as described previously (30).

### Treatment of NRK and COS-7 cells with NEM followed by immunofluorescence

NRK cells were plated onto glass cover slips and washed twice with HEPES buffer (20 mmHEPES, 137 mmNaCl, 3 mmKCl, 1 mmCaCl_2_, 2 mmMgCl_2_, 1 mg/mL glucose and 1 mg/ml BSA) and treated with 100 μmNEM in HEPES buffer for 5 min at 37^o^C. Twenty micromolar β-ME was added immediately to quench the NEM. The cells were washed twice with PBS. One set of cells were fixed with 4% paraformaldehyde and subsequently permeabilized with digitonin (40 μg/mL) for 10 min on ice and washed with cold PBS. A second set of cells were permeabilized with digitonin (40 μg/mL) for 10 min on ice and washed with cold PBS before fixation with paraformaldehyde. The cells were incubated with primary antibodies [mAb 4A7 for PITPβ and GM130 (polyclonal)] as indicated, followed by fluorescent Alexa fluor 488 (PITPβ) or 546 (GM130) conjugated secondary antibodies (Molecular Probes). Fluorescence was recorded by excitation at 488 or 546 nm with a light source (excite 120 nm) using an Olympus IX80 microscope fitted with a ×100 or a ×40 oil immersion objective. Images were acquired with a charge-coupled device camera ORCA-ER (Hamamatsu) cooled to −35°C and controlled with the Cell ^ F software (Olympus).

COS-7 cells were transfected with rat PITPβ-WT and the corresponding WW203/204AA mutant using FuGene 6 transfection reagent as per manufacturer’s recommendations (Roche Diagnostics). After 48 h, the cells were treated with 100 μmNEM for 2 min at 37°C. The cells were prepared exactly as above for microscopy except that the cells were fixed with 4% paraformaldehyde and subsequently permeabilized with digitonin (40 μg/mL) for 10 min on ice and washed with cold PBS. The cells were incubated with primary antibodies [mAb 4A7 for PITPβ and Giantin (rabbit polyclonal)] as indicated, followed by fluorescent Alexa fluor 488 (PITPβ) or 546 (Giantin) conjugated secondary antibodies (Molecular Probes).

## Supporting Information

Figure S1 Expression levels of PITPa and PITPb in cultured cell lines Lysates from cell lines were prepared in Radio Immuno Precipitation Assay buffer (RIPA) (50 mM Tris, pH 7.5; 150 mM NaCl; 1% NP-40; 0.5% deoxycholate and 0.1% sodium dodecyl sulphate) with the addition of protease inhibitor cocktail (Sigma). Lysates were centrifuged at 15 000 × g for 30 min at 4°C to remove cell debris, and proteins were separated by SDS–PAGE, followed by western blot analysis using isoform-specific antibodies. PITPα and PITPβ were quantified by western blot using recombinant PITPα and PITPβ-sp1 as standards. PITPβ was detected with Ab 1C1, which detects both splice variants (21). On each gel, a known concentration range of recombinant PITP proteins were included and the amount of PITP proteins present in the sample calculated based from them. Cell lines used were HL60 cells, human promyelocytic leukaemia; PC12 cells, derived from a rat pheochromocytoma, a tumour of the adrenal gland; NRK, normal rat kidney epithelial cells; RBL-2H3, rat basophilic leukaemia; WKPT-02903 CL2, immortalized from the Wistar rat proximal convoluted tubule; HeLa, human epithelial cell derived from a cervical tumour; COS-7, African green monkey kidney fibroblast-like cell line.

Figure S2 Inhibition of PtdIns transfer activity of PITPβ and its retention to membranes depend on NEM concentration and incubation time HL60 cells were treated with the indicated concentrations of NEM for 10 min (A and B) or 2 min (C and D). Membranes and cytosol were prepared. PtdIns transfer activity in control and NEM-treated HL60 cell cytosol (100 μg protein per assay) was assessed (A and C). B) Immunoblot of the membranes and cytosol fractions from (A) using an anti-PITPβ antibody, Ab 1C1. D) Immunoblot of PITPβ in membrane fractions treated with a range of NEM concentrations for 2 min.

Figure S3 NEM treatment retains PITPβ at the Golgi and ER compartment in NRK cells NRK cells were treated with 100 μm NEM for 5 min, quenched with β-ME (20 mm), permeabilized with digitonin (40 μg/mL) on ice and subsequently fixed with 4% paraformaldehyde. Endogenous PITPβ was revealed by immunofluorescence using the rat-specific anti-PITPβ monoclonal antibody 4A7 and the Golgi by antibody to GM130 (A–C) control NRK cells; (D–F) NRK cells were treated with 100 μm NEM for 5 min. A and D) PITPβ, green; B and E) GM130, red; C and F) Overlay of PITPβ and GM130. Bar scale: 10 μm.

Figure S4 Inhibition of PtdIns transfer activity by NEM is dependent on Cys95 Cys95 was mutated to alanine or threonine, and cys188 was mutated to alanine. PITPα and PITPβ mutants were purified as described in the *Materials and Methods*. A and C) One microgram of each protein was analysed by SDS–PAGE and Coomassie staining to examine their purity; B and D) PITP proteins (WT and mutants) were assayed for PtdIns transfer in the presence and absence of NEM (5 mm). A and B) PITPβ and its mutants and C and D) PITPα and its mutants.

Figure S5 NEM can only inhibit PtdIns transfer activity provided that membranes and PITP proteins are both present during the assay The recombinant proteins were assayed for PtdIns transfer after the following treatments. Filled circles, PITP protein incubated with varying concentrations of NEM in the absence of membranes; NEM was subsequently quenched with β-ME prior to assay for PtdIns transfer assay; Open circles, PITP proteins assayed for transfer activity in the presence of NEM with no pretreatment. Filled squares, membranes incubated with varying concentrations of NEM; NEM was quenched with β-ME, and the membranes were used as the donor compartment for PtdIns transfer. A) PITPβ and B) PITPα.
