# Supplementary figures and images for: Dynamics of Lipid Transfer by Phosphatidylinositol Transfer Proteins in Cells
Source: Traffic. 2008 Aug 6;9(10):1743–56. doi: 10.1111/j.1600-0854.2008.00794.x (PMC2635478; doi:10.1111/j.1600-0854.2008.00794.x)

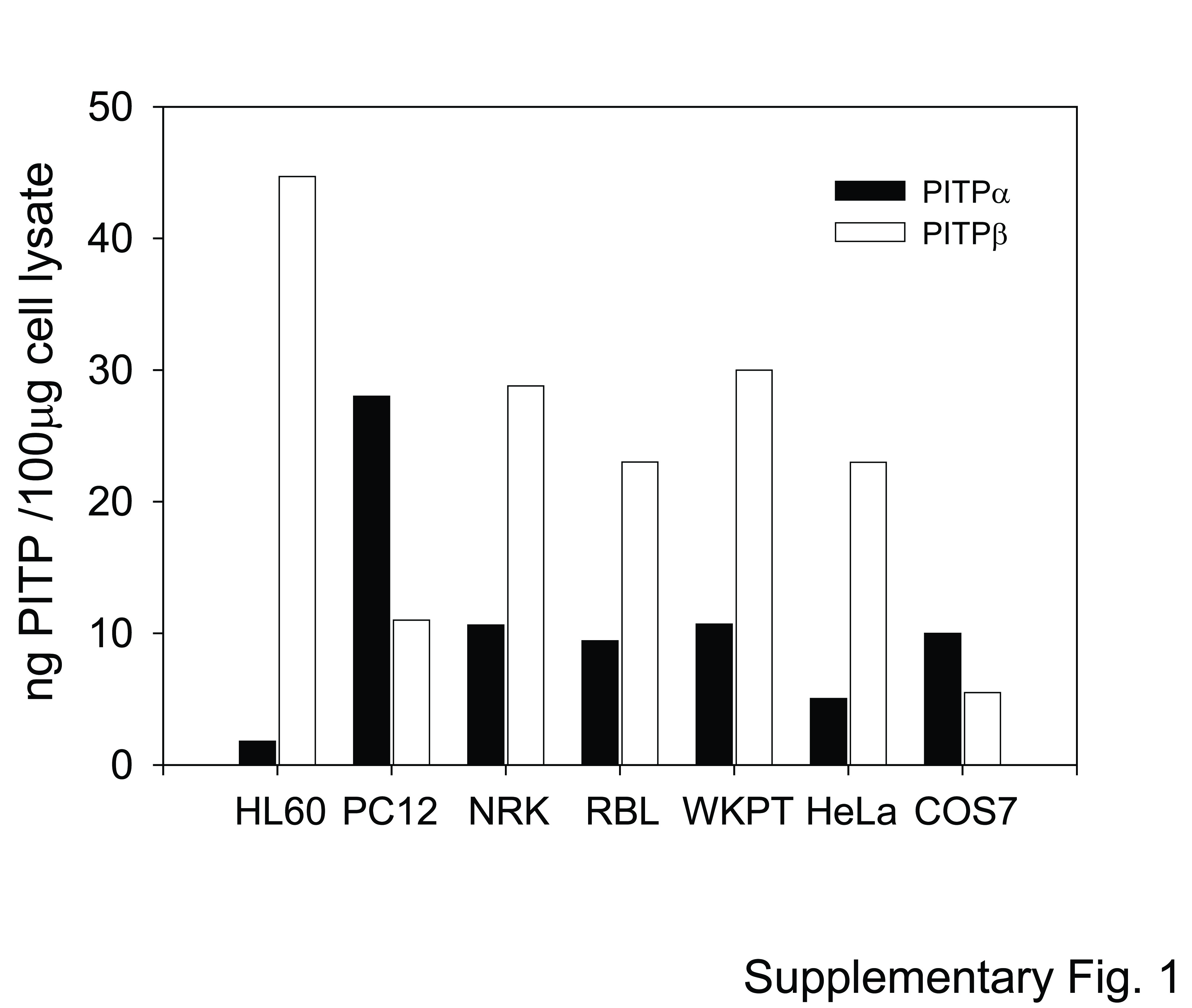

Supplement: Figure S1 — Lysates from cell lines were prepared in Radio Immuno Precipitation Assay buffer (RIPA) (50 mM Tris, pH 7.5; 150 mM NaCl; 1% NP-40; 0.5% deoxycholate and 0.1% sodium dodecyl sulphate) with the addition of protease inhibitor cocktail (Sigma). Lysates were centrifuged at 15 000 × g for 30 min at 4°C to remove cell debris, and proteins were separated by SDS–PAGE, followed by western blot analysis using isoform-specific antibodies. PITPα and PITPβ were quantified by western blot using recombinant PITPα and PITPβ-sp1 as standards. PITPβ was detected with Ab 1C1, which detects both splice variants (21). On each gel, a known concentration range of recombinant PITP proteins were included and the amount of PITP proteins present in the sample calculated based from them. Cell lines used were HL60 cells, human promyelocytic leukaemia; PC12 cells, derived from a rat pheochromocytoma, a tumour of the adrenal gland; NRK, normal rat kidney epithelial cells; RBL-2H3, rat basophilic leukaemia; WKPT-02903 CL2, immortalized from the Wistar rat proximal convoluted tubule; HeLa, human epithelial cell derived from a cervical tumour; COS-7, African green monkey kidney fibroblast-like cell line. [file tra0009-1743-SD1.jpg]

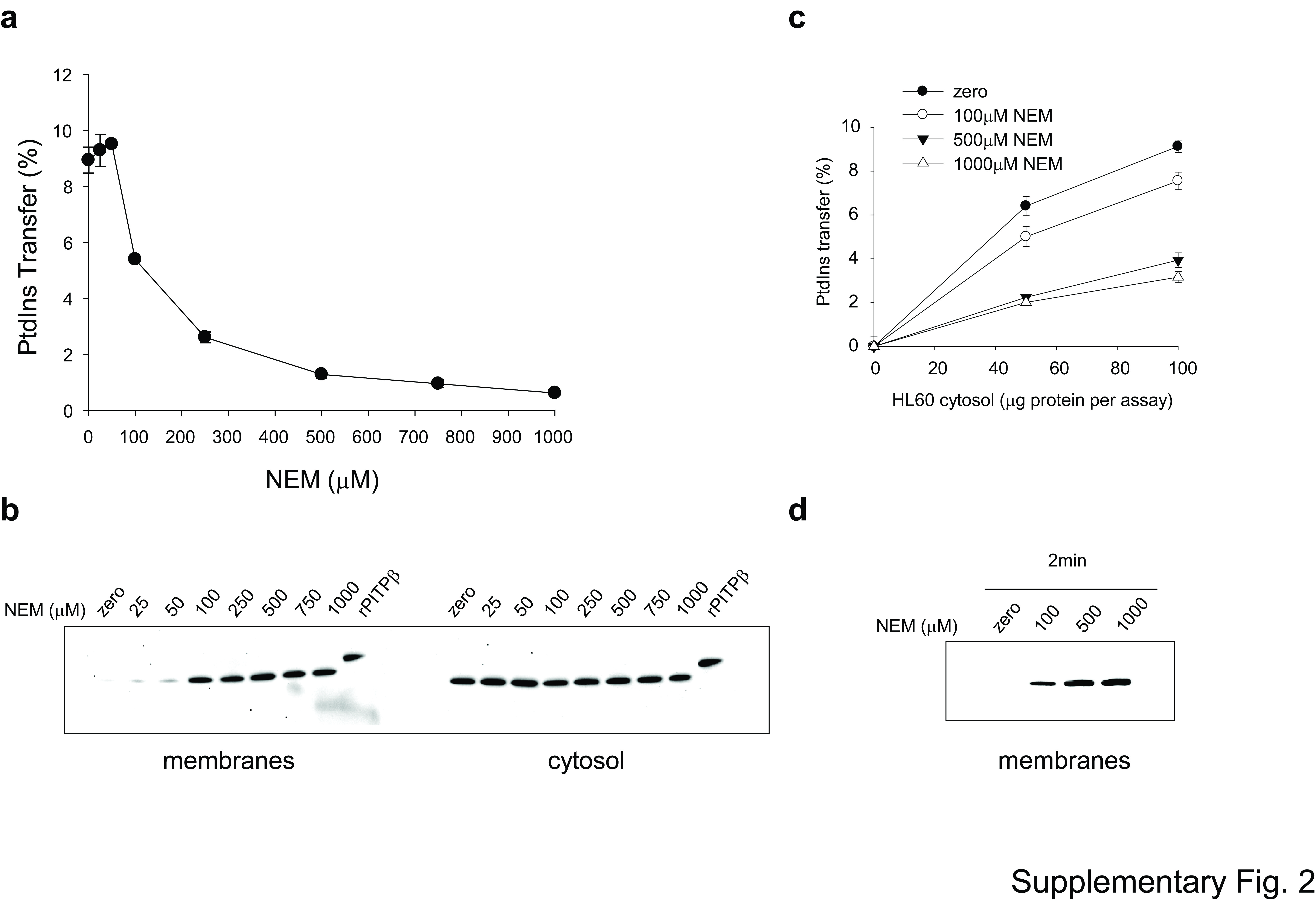

Supplement: Figure S2 — HL60 cells were treated with the indicated concentrations of NEM for 10 min (A and B) or 2 min (C and D). Membranes and cytosol were prepared. PtdIns transfer activity in control and NEM-treated HL60 cell cytosol (100 μg protein per assay) was assessed (A and C). B) Immunoblot of the membranes and cytosol fractions from (A) using an anti-PITPβ antibody, Ab 1C1. D) Immunoblot of PITPβ in membrane fractions treated with a range of NEM concentrations for 2 min. [file tra0009-1743-SD2.jpg]

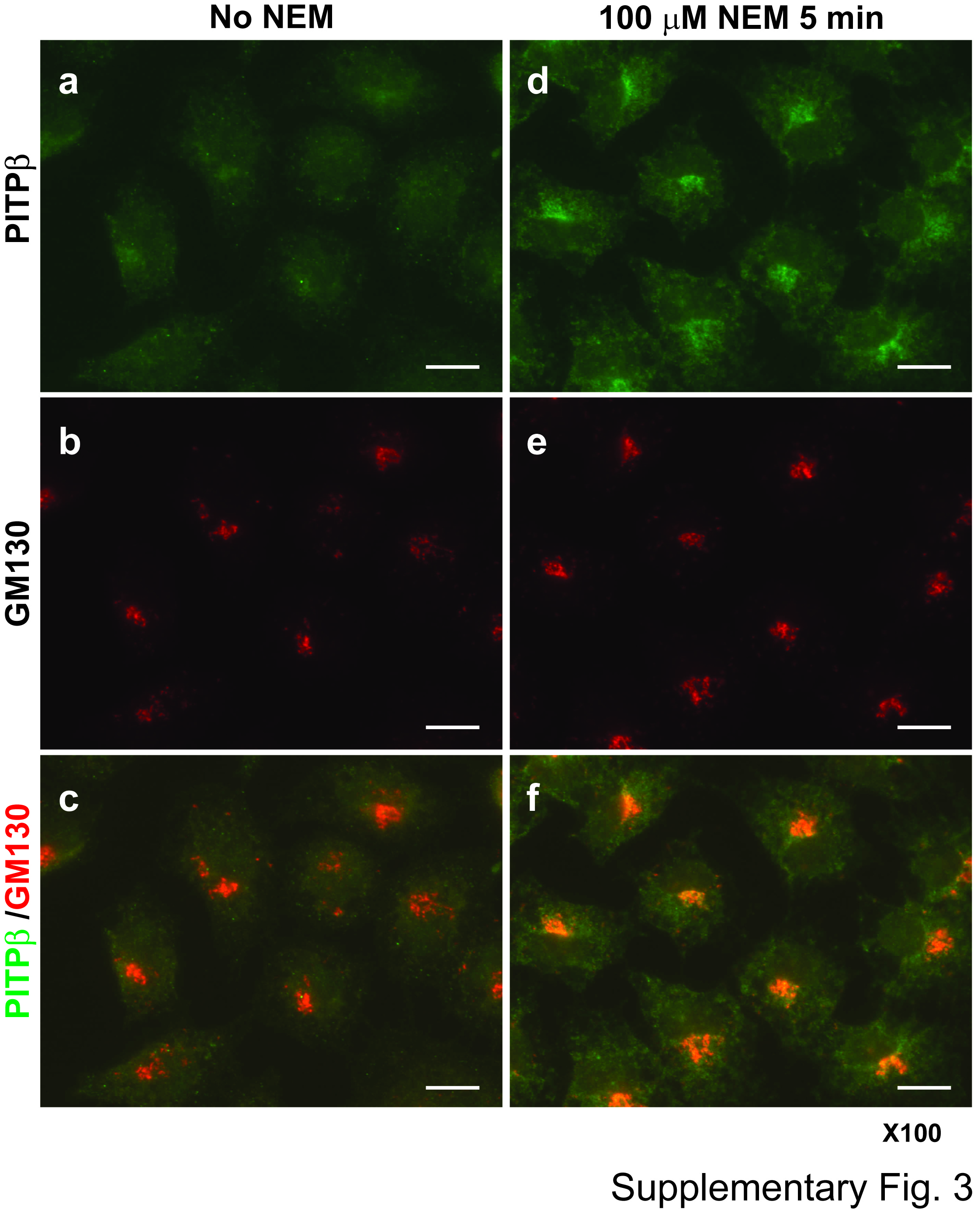

Supplement: Figure S3 — NRK cells were treated with 100 μm NEM for 5 min, quenched with β-ME (20 mm), permeabilized with digitonin (40 μg/mL) on ice and subsequently fixed with 4% paraformaldehyde. Endogenous PITPβ was revealed by immunofluorescence using the rat-specific anti-PITPβ monoclonal antibody 4A7 and the Golgi by antibody to GM130 (A–C) control NRK cells; (D–F) NRK cells were treated with 100 μm NEM for 5 min. A and D) PITPβ, green; B and E) GM130, red; C and F) Overlay of PITPβ and GM130. Bar scale: 10 μm. [file tra0009-1743-SD3.jpg]

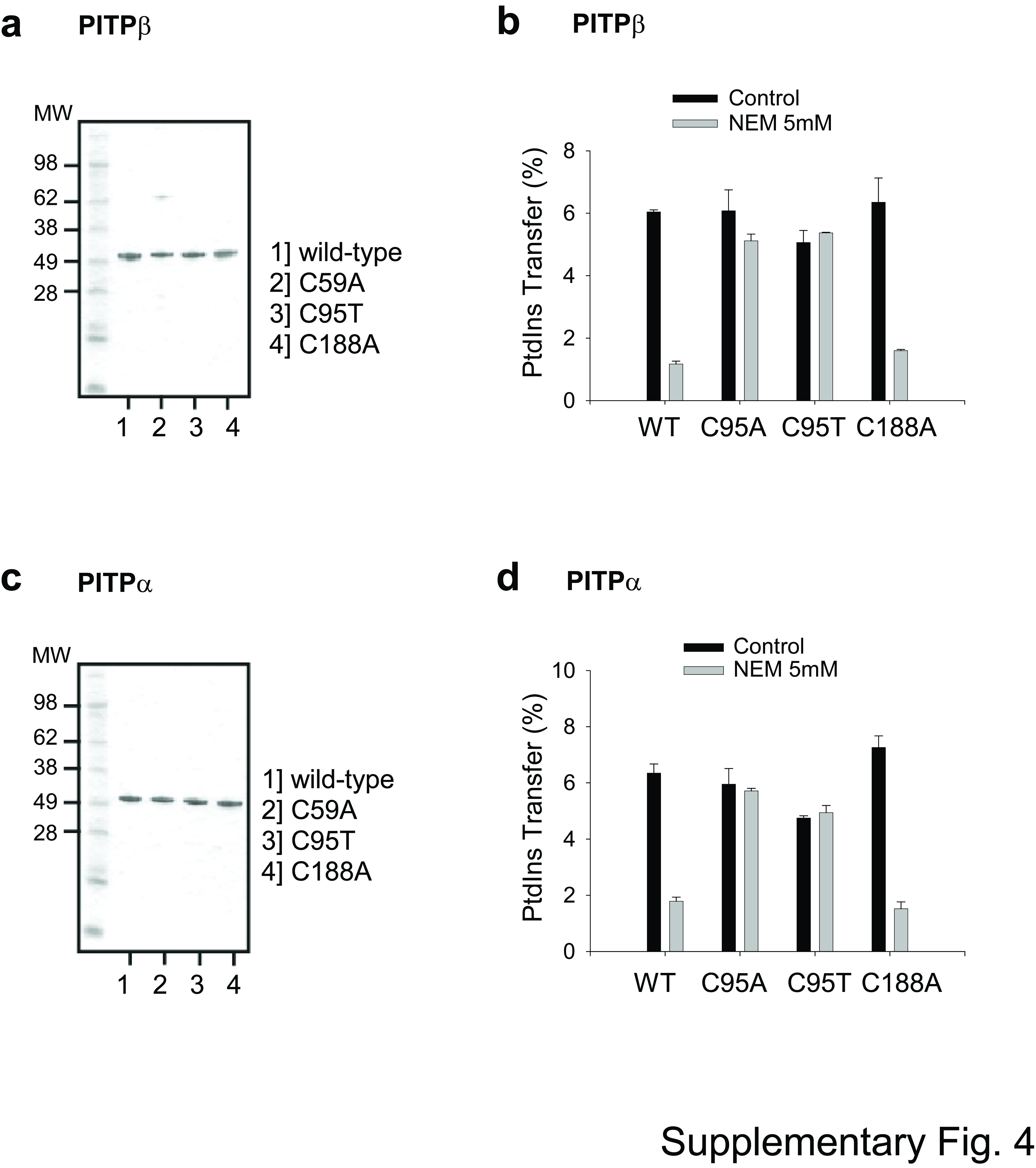

Supplement: Figure S4 — Cys95 was mutated to alanine or threonine, and cys188 was mutated to alanine. PITPα and PITPβ mutants were purified as described in the Materials and Methods. A and C) One microgram of each protein was analysed by SDS–PAGE and Coomassie staining to examine their purity; B and D) PITP proteins (WT and mutants) were assayed for PtdIns transfer in the presence and absence of NEM (5 mm). A and B) PITPβ and its mutants and C and D) PITPα and its mutants. [file tra0009-1743-SD4.jpg]

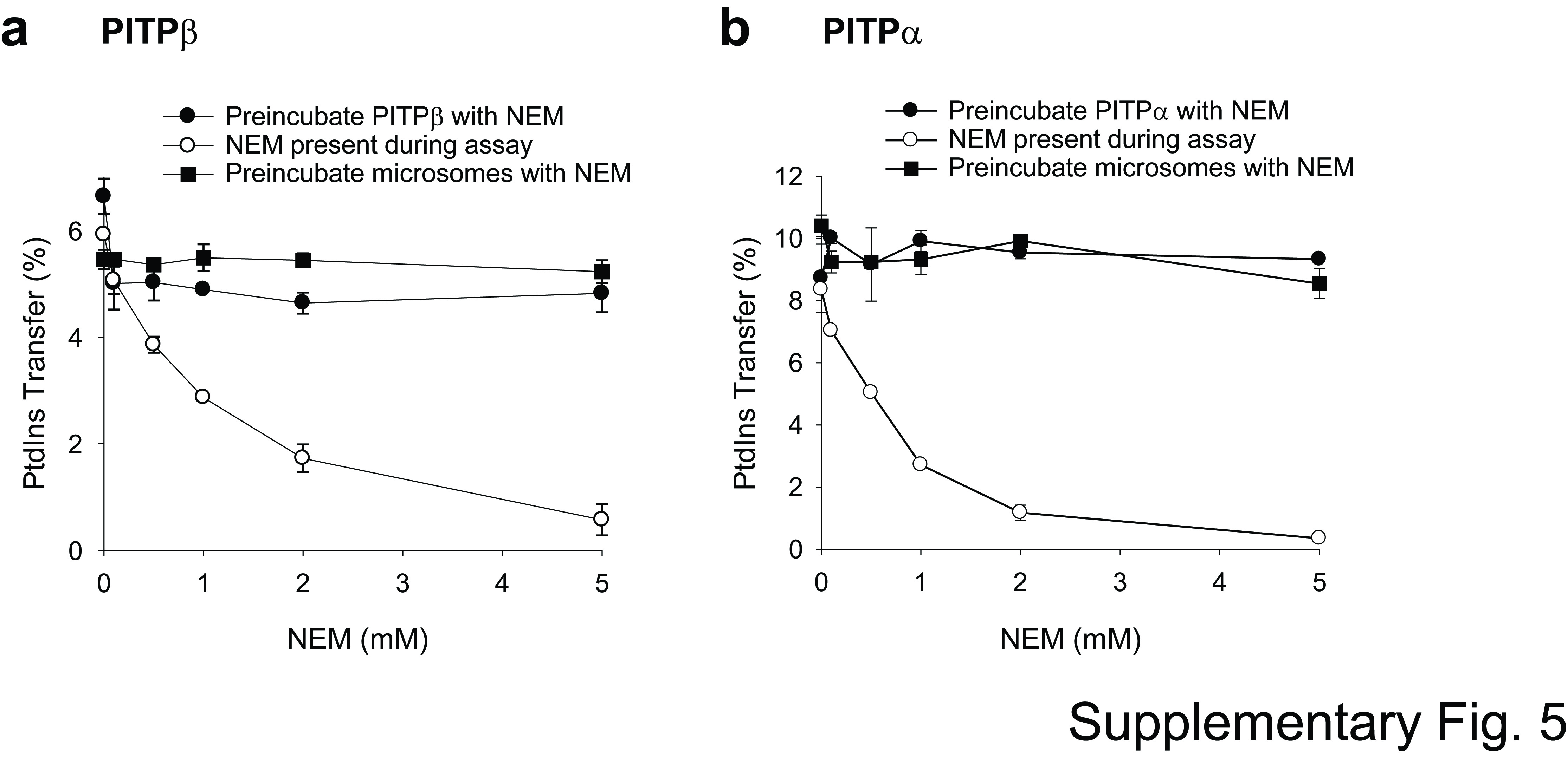

Supplement: Figure S5 — The recombinant proteins were assayed for PtdIns transfer after the following treatments. Filled circles, PITP protein incubated with varying concentrations of NEM in the absence of membranes; NEM was subsequently quenched with β-ME prior to assay for PtdIns transfer assay; Open circles, PITP proteins assayed for transfer activity in the presence of NEM with no pretreatment. Filled squares, membranes incubated with varying concentrations of NEM; NEM was quenched with β-ME, and the membranes were used as the donor compartment for PtdIns transfer. A) PITPβ and B) PITPα. [file tra0009-1743-SD5.jpg]
